# Quick assessment with controlled attenuation parameter for hepatic steatosis in children based on MRI-PDFF as the gold standard

**DOI:** 10.1186/s12887-019-1485-8

**Published:** 2019-04-15

**Authors:** Jaeseung Shin, Myung-Joon Kim, Hyun Joo Shin, Haesung Yoon, Seung Kim, Hong Koh, Mi-Jung Lee

**Affiliations:** 1 Department of Radiology and Research Institute of Radiological Science, Severance Children’s Hospital, Yonsei University College of Medicine, 50-1Yonsei-ro, Seodaemun-gu, Seoul, 03722 South Korea; 2 Severance Pediatric Liver Disease Research Group, Severance Children’s Hospital, Yonsei University College of Medicine, Seoul, South Korea; 3 Department of Pediatrics, Severance Children’s Hospital, Yonsei University College of Medicine, Seoul, South Korea

**Keywords:** Fatty liver, Non-alcoholic fatty liver disease, Children, Controlled attenuation parameter, Proton density fat fraction

## Abstract

**Background:**

Controlled attenuation parameter (CAP) is a recently introduced, non-invasive and quantitative method to evaluate hepatic steatosis demonstrated in adults, but limited in obesity and not well evaluated in children. The aim of this study was to investigate the diagnostic performance for assessing hepatic steatosis grades using CAP in children based on MR proton density fat fraction (PDFF).

**Methods:**

Children evaluated for non-alcoholic fatty liver disease (NAFLD) who were assessed for PDFF and CAP were enrolled retrospectively. Hepatic steatosis grades 0–3 were classified according to PDFF using cutoff values of 6, 17.5, and 23.3%. Subgroup analyses were performed in non-obese and obese groups using the 95th percentile body mass index (BMI) as a cutoff and BMI30 group when BMI > 30 kg/m^2^. Pearson’s correlations between variables were also analyzed.

**Results:**

In a total of 86 children, there were 53 in the obese group including 17 of the BMI30 group. CAP demonstrated 98.7% sensitivity and 80% specificity for diagnosing grades 1–3 vs. grade 0 using a cutoff value of 241 dB/m (area under the curve = 0.941, *p* < 0.001). The diagnostic performance for higher steatosis grades was suboptimal. CAP correlated with abdominal wall thickness in both obese (r = 0.549, *p* = 0.001) and non-obese (r = 0.386, *p* = 0.004) groups and did not correlate with PDFF in BMI30 group.

**Conclusion:**

In children with NAFLD, CAP showed excellent diagnostic performance for differentiating presence and absence of hepatic steatosis using a cutoff value of 241 dB/m. However, CAP was limited in evaluating grades of steatosis, especially in children with BMI > 30 kg/m^2^.

## Background

Non-alcoholic fatty liver disease (NAFLD) is the most prevalent liver disease in children [1]. It has a large spectrum of presentation, can progress, and is associated with dyslipidemia, diabetes, and cardiovascular disease [2]. The prevalence of NAFLD was reported by race and ethnicity among a pediatric population as: Asian: 10.2%, Black: 1.5%, Hispanic: 11.8%, and White: 8.6% [1]. With worldwide increasing trends of obesity and consequently NAFLD in children and adolescents, including South Korea as the prevalence of obesity increased from 6.8% in 1998 to 10.0% in 2013 [3, 4], there is increased risk of liver, cardiovascular and metabolic diseases throughout the lifespan. Although liver biopsy is the clinical standard for diagnosis, it is an invasive procedure with sampling errors and questionable inter- and intra-observer reliability [5]. Therefore, liver biopsy may not be ideal for all patients suspected of having NAFLD and for longitudinal follow-up, especially in children.

Non-invasive liver imaging techniques for hepatic steatosis have been emerging as a substitute for liver biopsy. MRI-estimated liver proton density fat fraction (PDFF) has shown a good correlation with histologic steatosis grade and the potential of clinical utility for the evaluation of NAFLD in both adults and children [6, 7]. Moreover, it not only has a high precision and reproducibility, but also greater reliability than histologic grading [8]. In a multicenter study for children with NAFLD, PDFF has shown high diagnostic accuracy to classify and predict histological steatosis grade, as well as to monitor changes in steatosis [9]. However, widespread use of PDFF might be limited in pediatric clinics due to high cost with need for expertise and longer examination time.

Transient elastography (TE) is an ultrasound-based technology used to estimate quantitative liver elasticity and is commonly used in clinical practice [10]. Controlled attenuation parameter (CAP), a novel technique to estimate hepatic steatosis using ultrasound attenuation based on the TE, shows correlation with histologic grades in adults [11–13]. Previous studies in pediatric liver disease have also shown encouraging outcomes for assessing steatosis using CAP [14]. CAP has advantages over MRI in terms of cost, accessibility, and quick assessment. However, the lack of optimal cutoff values for hepatic steatosis and technical limitation in obese patients [15], which is a risk factor for NAFLD [16], still remain in pediatric patients.

Therefore, in the present study, we aimed to evaluate the diagnostic performance of CAP for assessing hepatic steatosis in children based on PDFF with subgroup analyses based on body mass index (BMI).

## Methods

### Patients

This retrospective study was approved by the Institutional Review Board of our hospital. The acquisition of informed consent was waived. Pediatric patients aged 18 years or younger who underwent both abdominal MRI including PDFF and TE with CAP as a routine clinical practice for the evaluation of NAFLD in our hospital were included in this study. We included only the examinations within one month interval from January 2015 to December 2016. We excluded patients who had clinical or laboratory evidence of a liver diagnosis other than NAFLD (e.g., glycogen storage disease, drug, or virus) or alcohol consumption. We also reviewed laboratory results including aspartate aminotransferase (AST) and alanine aminotransferase (ALT) levels. Patients were divided into non-obese and obese groups based on BMI using the age and sex dependent 95th percentile as the cutoff [17]. We also classified patients with a BMI greater than 30 kg/m^2^ among the obese group as a separate BMI30 group for additional analysis.

### Liver MRI including PDFF

All MR scans were performed with patients lying supine in a 3-T scanner (Discovery MR750, GE Healthcare, Waukesha, WI, USA) with a 32-channel body coil without sedation. MR acquisition included single shot fast spin echo (SSFSE) T2-weighted axial and coronal images and iterative decomposition of water and fat with echo asymmetry and least-squares estimation quantification (IDEAL-IQ) axial images of the liver. SSFSE was used to identify anatomical locations and lesions in the liver as well as to measure abdominal wall thickness (AWT), which was defined as the thinnest skin-to-liver capsule distance of the abdominal wall surrounding the liver on an axial image at the main portal vein level. The IDEAL-IQ sequence is a three-dimensional volumetric imaging sequence for creating water, fat, in-phase, out-of-phase, R2* (1/T2*), and fat fraction (water-triglyceride fat separation) maps of the liver from a single breath hold acquisition. The parameters of IDEAL-IQ were as follows: repetition time, 5.8 msec; field of view, 35–42 cm; bandwidth, 125 kHz; flip angle, 3°; section thickness, 8 mm; and a single three-dimensional image with 25 to 30 sections.

PDFF measurements in IDEAL-IQ were performed by placing regions of interest (ROIs) with the maximal area in the right hemiliver in three contiguous images. The ROIs were oval or circular in shape and excluded the liver boundary, fissures, gall bladder fossa, artifacts, and large blood vessels. Finally, the average value of the three measurements was used as the representative value. Patients’ steatosis grades were grouped according to the established PDFF cutoff values for diagnosing histological steatosis grades 1 (S1), 2 (S2), and 3 (S3), and the PDFF cutoff values used were 6% for S1, 17.5% for S2, and 23.3% for S3 [9, 18].

### TE for CAP

CAP measurements were performed using Fibroscan (Echosens, France) by experienced technicians. TE was performed on the right lobe of the liver through the intercostal space with the participant lying supine with the right arm in maximal abduction. All participants underwent TE using an M or XL probe according to body size. The M probe was used for patients with a thoracic perimeter more than 75 cm, and the XL probe was used when the distance from the skin to the liver capsule was estimated over 25 mm. CAP measured ultrasonic attenuation at 3.5 MHz in the M probe and 2.5 MHz in the XL probe using signals that were acquired from TE. The median value of 10 valid measurements for a given participant was selected as the representative CAP value.

### Statistical analyses

Statistical analyses were performed using the SPSS software package (IBM SPSS Statistics version 21; IBM Corp., Armonk, NY, USA) and MedCalc software package (version 18.2.1; MedCalc Software, Ostend, Belgium). Pearson’s correlation coefficient (r) was calculated to evaluate the correlations between variables. To determine statistically significant differences in continuous variables between the non-obese and obese BMI groups, we used the one-way analysis of covariance with sex and age as covariates. For the comparison of PDFF and CAP in the BMI30 group, Mann Whitney test was used. According to steatosis groups based on PDFF, between-group differences were assessed by means of the Kruskal-Wallis test. Bonferroni’s correction was applied to the post hoc analysis of the between-group comparison. Differences in categorical variables were evaluated using the chi-square test or Fisher’s exact test as appropriate. Correlation coefficients between groups were compared with Fisher’s r-to-z transformation. Receiver-operating curve (ROC) analysis was used to evaluate the diagnostic performance of CAP for each steatosis grade. The optimal cutoff values were selected to maximize the Youden index. A *p*-value < 0.05 was considered statistically significant.

## Results

### Patient characteristics

Table 1 shows the characteristics and laboratory results of all study patients and each subgroup. A total of 86 patients (M: F = 62: 24) with a mean age of 13.1 ± 2.7 years (range, 7–18 years) were included in this study. The time interval between MRI and TE was 0–19 days with the mean of 2.4 ± 5.0 days. Among the included patients, 33 were classified in the non-obese group, and the remaining 53 patients belonged to the obese group. Seventeen patients also met the criteria for inclusion in the BMI30 group.

**Table 1:** Patient characteristics including comparison between patients with a non-obese body mass index (BMI) (non-obese group) and a BMI greater than the 95th percentile (obese group)

	All patients (*n* = 86)	Group comparison		
		Non-obese group (*n* = 33)	Obese group (*n* = 53)	*p*-value
Age (y)	13.1 ± 2.7	12.4 ± 2.4	13.5 ± 2.8	0.067
Female (n, %)	24 (31.3)	12 (36.4)	12 (22.6)	0.168^*^
BMI (kg/m^2^)	26.3 ± 4.9	21.6 ± 2.6	29.2 ± 3.5	**< 0.001**
AWT (cm)	2.5 ± 0.7	2.1 ± 0.5	2.8 ± 0.6	**< 0.001**
AST (U/L)	61.4 ± 49.7	46.2 ± 39.8	70.9 ± 53.2	**0.015** ^†^
ALT (U/L)	100.7 ± 85.2	71.2 ± 66.9	119.0 ± 90.7	**0.004** ^†^
Total bilirubin (mg/dL)	0.6 ± 0.3	0.5 ± 0.2	0.6 ± 0.3	0.861
Albumin (g/dL)	4.6 ± 0.3	4.6 ± 0.3	4.6 ± 0.2	0.702
ALP (IU/L)	206.5 ± 100.5	222.6 ± 99.0	196.5 ± 101.0	0.728
Cholesterol (mg/dL)	190.7 ± 35.9	185.8 ± 38.0	193.8 ± 34.5	0.229
TG (mg/dL)	159.5 ± 90.4	151.0 ± 86.0	163.3 ± 93.2	0.888^†^
HDL (mg/dL)	44.3 ± 10.0	45.8 ± 12.2	43.5 ± 8.7	0.501
LDL (mg/dL)	117.1 ± 34.4	113.9 ± 38.8	118.6 ± 32.3	0.356
PDFF (%)	22.6 ± 12.8	20.2 ± 14.5	24.1 ± 11.6	0.139
CAP (dB/m)	310.5 ± 46.5	293.6 ± 51.5	321.1 ± 40.2	0.053

* Chi-square test was performed to compare the two groups

^†^Logarithmic transformation before group comparison was performed in order to satisfy normality assumption. All the *p*-values of boldface are less than 0.05

Notes: *AWT* abdominal wall thickness, *AST* aspartate aminotransferase, *ALT* alanine aminotransferase, *ALP* alkaline phosphatase, *TG* triglycerides, *HDL* high-density lipoprotein, *LDL* low-density lipoprotein, *PDFF* proton density fat fraction, *CAP* controlled attenuation parameter

There was no significant difference in age or gender between the non-obese and obese groups; however, AWT was greater in the obese group compared to the non-obese group (2.8 ± 0.6 cm vs. 2.1 ± 0.5 cm, *p* < 0.001). The mean AST (70.9 ± 53.2 IU/L vs. 46.2 ± 39.8 IU/L, *p* = 0.025) and ALT (119.0 ± 90.7 IU/L vs. 71.2 ± 66.9 IU/L, *p* = 0.011) values were significantly higher in the obese group compared to the non-obese group. The logarithmic transformed AST (1.77 ± 0.28 vs. 1.61 ± 0.32, *p* = 0.015) and ALT (2.00 ± 0.29 vs. 1.72 ± 0.47, *p* = 0.004) values were also significantly different between the two groups. The other laboratory results were not significantly different between the two groups (Table 1).

There was a positive correlation between AWT and BMI (r = 0.807; 95% confidence interval [CI]: 0.718, 0.870). For the CAP assessment of TE, M and XL probes were used for eighty-two and four patients, respectively. The four patients used XL probe were all in the BMI30 group. Patient characteristics according to steatosis group based on PDFF are summarized in Table 2.

**Table 2:** Patient characteristics according to steatosis grades based on MR proton density fat fraction

	Steatosis grades				
	S0 (*n* = 10)	S1 (*n* = 25)	S2 (*n* = 14)	S3 (n = 37)	*p*-value^*^
Age (y)	13 (9–17)	14 (7–18)	13 (10–18)	12 (9–18)	0.209
Female (n, %)	5 (50.0)	8 (32.0)	5 (35.7)	6 (16.2)	0.134^†^
BMI (kg/m^2^)	20.6 (14.7–25.9)	26.3 (18.0–38.5)	26.5 (19.2–30.9)	28.3 (19.3–40.6)	**0.002**
AWT (cm)	1.7 (1–2.9)	2.7 (1.3–4.3)	2.5 (1.8–3.2)	2.8 (1.8–4.7)	**0.003**
AST (U/L)	21.5 (17–26)	52 (12–241)	36 (17–197)	63 (25–179)	**< 0.001**
ALT (U/L)	15 (8–28)	95 (8–430)	61 (22–234)	96 (23–346)	**< 0.001**
Total bilirubin (mg/dL)	0.6 (0.3–0.8)	0.6 (0.3–1.2)	0.5 (0.3–1.6)	0.5 (0.3–0.9)	0.089
Albumin (g/dL)	4.4 (4.2–5.1)	4.6 (3.7–5.2)	4.5 (4.0–4.9)	4.6 (4.3–5.1)	0.249
ALP (IU/L)	217 (69–325)	152 (38–405)	201 (59–308)	242 (65–466)	**0.032**
Cholesterol (mg/dL)	161.5 (127–231)	184 (144–306)	205 (151–222)	191 (125–276)	0.044
TG (mg/dL)	91 (44–419)	117 (58–240)	144.5 (55–322)	158 (54–487)	0.045
HDL (mg/dL)	50.5 (26–66)	39 (26–70)	44 (28–50)	47 (30–65)	0.133
LDL (mg/dL)	79.1 (60.2–106.8)	117.6 (71.2–235.8)	128 (90.8–183)	107.8 (66.6–205)	**0.010**
PDFF (%)	3.5 (2.6–5.2)	13.9 (6.8–17)	19.5 (17.8–23.1)	33.7 (24–48)	**< 0.001**
CAP (dB/m)	222.5 (157–311)	308 (216–400)	301 (271–384)	329 (272–399)	**< 0.001**

The values are median (range) or number (percentage)

*Non-parametric method, Kruskal-Wallis test, was performed to compare groups, unless otherwise indicated

^†^Chi-square test was performed to compare groups. All the *p*-values of boldface are less than 0.05

Notes: *AWT* abdominal wall thickness, *AST* aspartate aminotransferase, *ALT* alanine aminotransferase, *ALP* alkaline phosphatase, *TG* triglycerides, *HDL* high-density lipoprotein, *LDL* low-density lipoprotein, *PDFF* proton density fat fraction, *CAP* controlled attenuation parameter

### Diagnostic performance of CAP

**Fig. 1:**
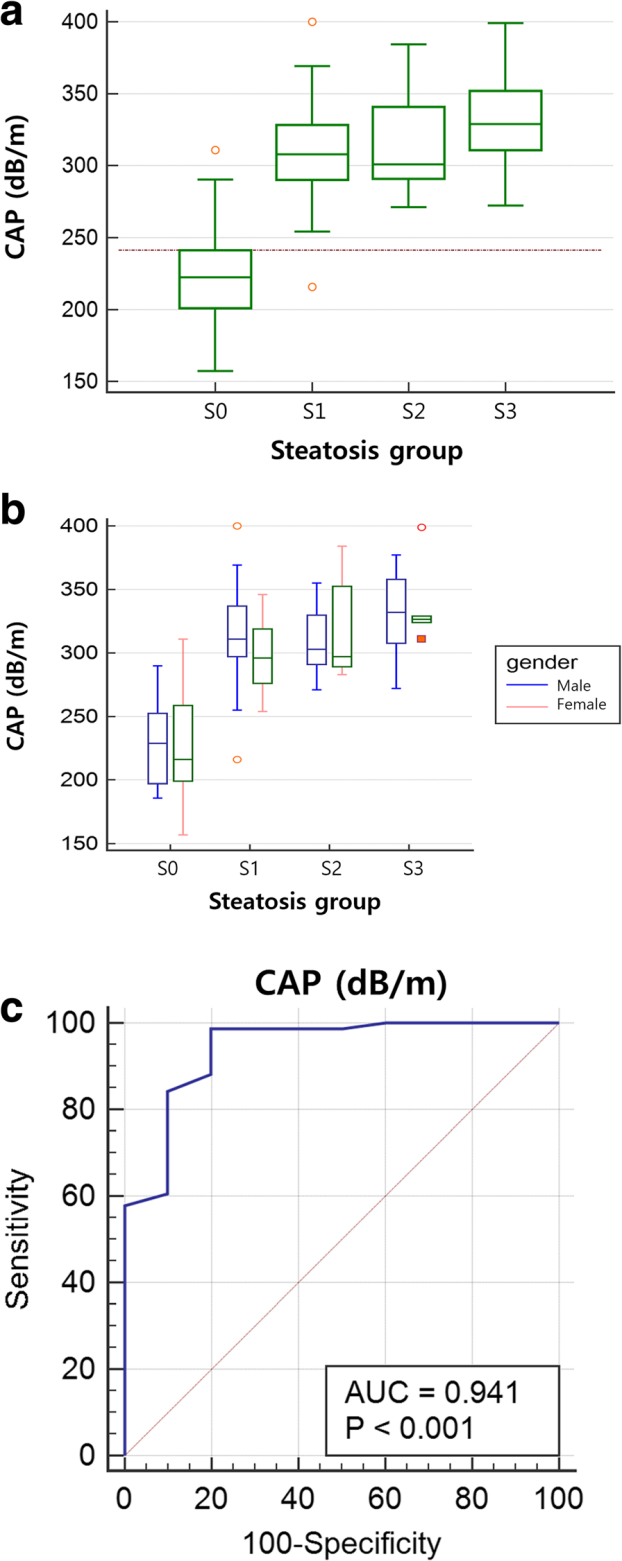
Comparison of steatosis groups using controlled attenuation parameter (CAP) value. (A and B) CAP values in each steatosis group based on MR proton density fat fraction (PDFF) of whole group (**a**) and divided by gender (**b**) are demonstrated in a box plot. Hepatic steatosis grades 0–3 (S0-S3) were classified using the PDFF cutoff values of 6, 17.5, and 23.3%. The mean and median CAP values were 228.4 and 222.5 dB/m in S0, 309 and 308 dB/m in S1, 313.2 and 301 dB/m in S2, and 332.7 and 329 dB/m in S3, respectively. The dash line means the cutoff value of 241 dB/m to differentiate S0 vs. S1-S3. (**c**) On receiver operating characteristic curve analysis, the optimal cutoff value for diagnosis of S1-S3 vs. S0 was 241 dB/m with 98.7% sensitivity (95% confidence interval [CI], 92.9–100.0), 80.0% specificity (95% CI, 44.4–97.5), and 0.941 of area under the curve (95% CI, 0.868–0.980)

Both PDFF and CAP values were measured in all patients. The PDFF values ranged from 2.6–48.0% with a mean of 22.6 ± 12.8%. The CAP values ranged from 157 to 400 dB/m with a mean of 310.5 ± 46.5 dB/m. According to PDFF, patients were divided into four steatosis groups, S0 (PDFF < 6%, *n* = 10), S1 (PDFF 6–17.4%, *n* = 25), S2 (PDFF 17.5–23.2%, *n* = 14), and S3 (PDFF ≥23.3%, *n* = 37). The CAP values in each steatosis group are shown in Fig. 1. The mean and median CAP values were 228.4 ± 45.9 dB/m and 222.5 dB/m in S0, 309.0 ± 38.9 dB/m and 308 dB/m in S1, 313.2 ± 33.1 dB/m and 301 dB/m in S2, and 332.7 ± 28.3 dB/m and 329 dB/m in S3, respectively. The mean CAP value of S0 showed a statistically significant difference from the other groups (*p* < 0.001), but there were no significant differences among S1, S2, and S3.

In ROC analysis, a CAP value of 241 dB/m represented an optimal cutoff value for diagnosis of S1–S3 vs. S0, with a sensitivity of 98.7% (95% CI: 92.9, 100.0), a specificity of 80.0% (95% CI: 44.4, 97.5), and area under the curve (AUC) of 0.941 (95% CI: 0.868, 0.980) (Table 3, Fig. 1). Positive predictive value (PPV) and negative predictive value (NPV) for the presence of steatosis on a CAP value of 241 dB/m was 96.2 and 87.5%, respectively. A CAP value of 213 dB/m showed 100% sensitivity (95% CI: 95.3, 100.0) and 40% specificity (95% CI: 12.2, 73.8), whereas a CAP value of 311 dB/m showed 57.9% sensitivity (95% CI: 46.0, 69.1) and 100% specificity (95% CI: 69.2, 100.0) to diagnose the presence of steatosis. The optimal CAP cutoff values of 299 dB/m and 303 dB/m were obtained to predict S2–S3 vs. S0–S1 (sensitivity 80.4% with 95% CI of 66.9–90.2, specificity 51.4% with 95% CI of 34.0–68.6, PPV 70.0%, NPV 65.4%, AUC 0.734 with 95% CI of 0.627–0.823) and S3 vs. S0–S2 (sensitivity 81.1% with 95% CI of 64.8–92.0, specificity 57.1% with 95% CI of 42.2–71.2, PPV 57.7%, NPV 79.4%, AUC 0.752 with 95% CI of 0.647–0.839), respectively. The AUC was higher for the diagnosis of S1–S3 vs. S0 compared with that of S2–S3 vs. S0–S1 (*p* = 0.011) and that of S3 vs. S0–S2 (*p* = 0.015). However, it was not different for the diagnosis of S2–S3 vs. S0–S1 and that of S3 or not (*p* > 0.999).

**Table 3:** Diagnostic performance of CAP for hepatic steatosis grades (S0-S3)

	Cutoff (dB/m)	Sensitivity (%, 95% CI)	Specificity (%, 95% CI)	PPV (%)	NPV (%)	AUC (95% CI)
S0 vs. S1-S3 (≥6%)	241	98.7 (92.9–100.0)	80.0 (44.4–97.5)	96.2	87.5	0.941 (0.868–0.980)
S0-S1 vs. S2-S3 (≥17.5%)	299	80.4 (66.9–90.2)	51.4 (34.0–68.6)	70.0	65.4	0.734 (0.627–0.823)
S0-S2 vs. S3 (≥23.3%)	303	81.1 (64.8–92.0)	57.1 (42.2–71.2)	57.7	79.4	0.752 (0.647–0.839)

Notes: CAP, controlled attenuation parameter; CI, confidence interval; PPV, positive predictive value; NPV, negative predictive value; AUC, area under the curve

### Relationships in different body habitus groups

**Fig. 2:**
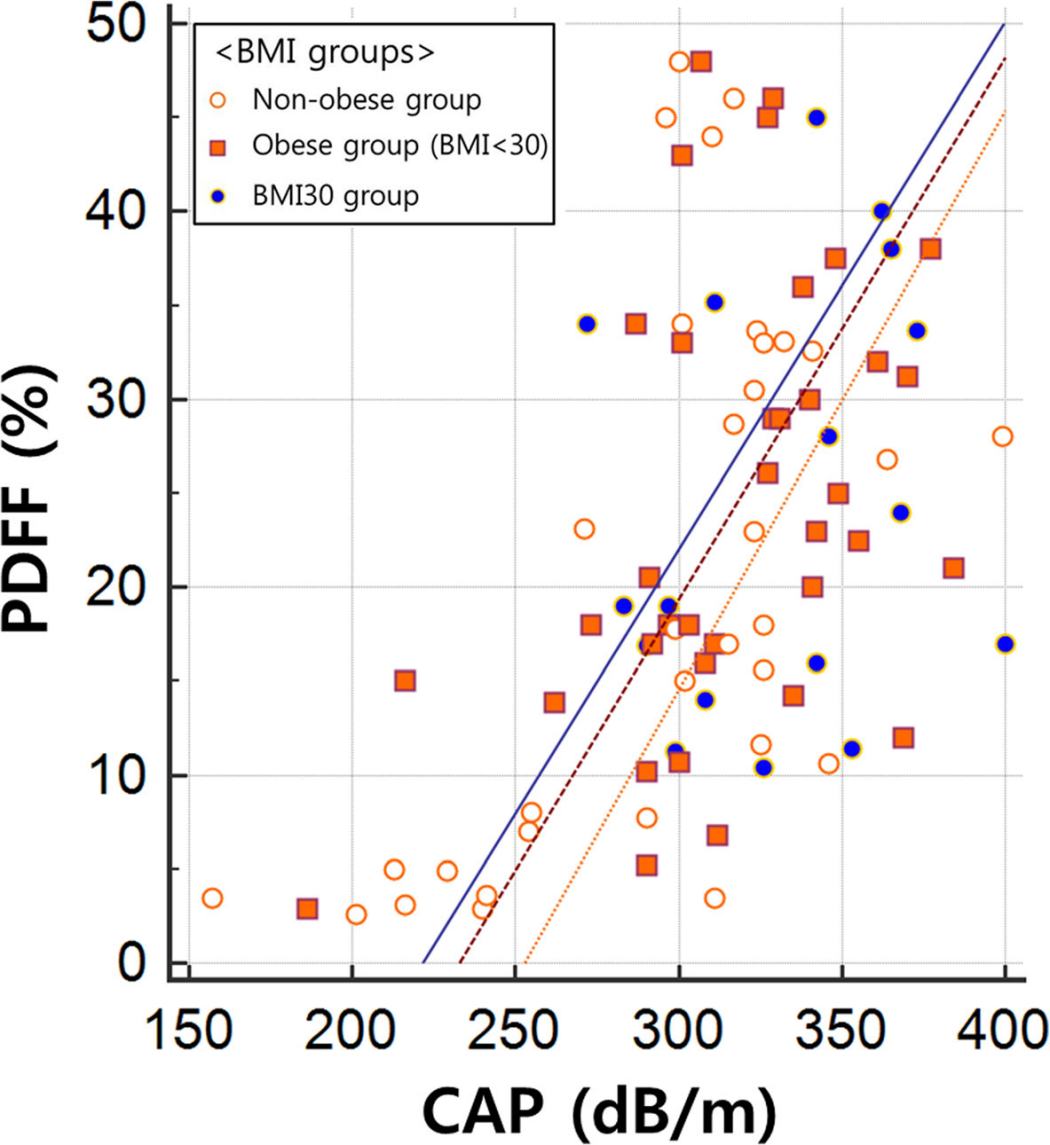
A scatter plot of hepatic steatosis between PDFF and CAP. CAP values were positively correlated with PDFF in all patients (r = 0.486; 95% confidence interval [CI]: 0.306, 0.633; *p* < 0.001). In subgroups according to body mass index (BMI), the r was 0.585 (95% CI: 0.302, 0.773; *p* < 0.001) in the non-obese group (BMI < 95th percentile) and 0.354 (95% CI: 0.093, 0.570; *p* = 0.009) in the obese group (BMI ≥ 95th percentile). However, PDFF and CAP values were not correlated in the BMI30 (BMI > 30 kg/m^2^) group

PDFF was positively correlated with CAP in all patients (r = 0.486; 95% CI: 0.306, 0.633; *p* < 0.001) (Table 4). According to subgroup analysis, the correlation coefficient between PDFF and CAP was 0.585 (95% CI: 0.302, 0.773) in the non-obese group (*n* = 33, *p* < 0.001) and 0.354 (95% CI: 0.093, 0.570) in the obese group (*n* = 53, *p* = 0.009), the difference of which was not statistically significant (z = 1.30, *p* = 0.097) (Fig. 2).

**Table 4:** Correlation values for hepatic steatosis in all patients, the non-obese group, obese group, and BMI30 (BMI > 30 kg/m^2^) group

		All patients (*n* = 86)		Non-obese group (*n* = 33)		Obese group (*n* = 53)		BMI30 group (*n* = 17)	
		r (95% CI)	*p*-value	r (95% CI)	*p*-value	r (95% CI)	*p*-value	r (95% CI)	*p*-value
PDFF	CAP	0.486 (0.306–0.633)	**< 0.001**	0.585 (0.302–0.773)	**< 0.001**	0.354 (0.093–0.570)	**0.009**	0.212 (− 0.300–0.629)	0.413
	AWT	0.179 (− 0.003–0.377)	0.099	0.318 (− 0.028–0.596)	0.071	−0.023 (− 0.300–0.249)	0.925	−0.338 (− 0.704–0.170)	0.185
CAP	AWT	0.517 (0.343–0.657)	**< 0.001**	0.549 (0.253–0.751)	**0.001**	0.386 (0.129–0.594)	**0.004**	0.221 (− 0.291–0.634)	0.394

Notes: *CI* confidence interval, *PDFF* proton density fat fraction, *CAP* controlled attenuation parameter, *AWT* abdominal wall thickness. All the *p*-values of boldface are less than 0.05

With respect to AWT, male (2.61 ± 0.63 cm) had significantly higher AWT than female did (2.39 ± 0.69; *p* = 0.045). PDFF was not correlated with AWT (Table 4). However, CAP was positively correlated with AWT (r = 0.517; 95% CI: 0.343, 0.657; *p* < 0.001). In subgroup analysis, only CAP was positively correlated with AWT in both the non-obese group (r = 0.549; 95% CI: 0.253, 0.751; *p* = 0.001) and obese group (r = 0.386; 95% CI: 0.129, 0.594; *p* = 0.004). AWT was not correlated with PDFF in either the non-obese or obese group.

In the BMI30 group, CAP values were obtained using M probe in 13 patients and XL probe in four patients. The median values of the PDFF and CAP in BMI30 group patients were 19 and 20% in PDFF, and 326 dB/m and 370 dB/m in CAP, obtained with M and XL probes, respectively. PDFF was not correlated with CAP (r = 0.212; 95% CI: -0.300, 0.629; *p* = 0.413). AWT was not correlated with PDFF (r = − 0.338; 95% CI: -0.704, 0.170; *p* = 0.185) and CAP (r = 0.221; 95% CI: -0.291, 0.634; *p* = 0.394).

## Discussion

NAFLD is becoming increasingly recognized as an important health problem for pediatric patients [19]. Because there is no established effective therapy, early risk stratification for disease progression is considered an important component of patient management [20]. In this retrospective study, we compared values for hepatic steatosis in pediatric NAFLD cases obtained noninvasively using PDFF and CAP techniques. Based on ROC analysis for diagnosing hepatic steatosis grades using established PDFF cutoff values, we suggest a CAP cutoff value of 241 dB/m for the presence of steatosis. However, CAP cutoff values for steatosis grade 2 or higher were not reliable. Although there were moderate correlations between PDFF and CAP values in all patients (r = 0.486), there was no correlation between PDFF and CAP in the BMI30 group. CAP values were positively correlated with AWT. Therefore, CAP can be a good screening tool to diagnose the presence of steatosis in children, but is probably limited during disease follow up or in children with high BMI, though longitudinal data are lacking.

MRI-based hepatic fat quantification is useful in pediatric patients not only for diagnosis and grading [9], but also for treatment monitoring [21]. PDFF exhibits an excellent correlation with hepatic steatosis, especially the macrovesicular form, which is common in both adult and pediatric NAFLD [22, 23]. PDFF also quantifies steatosis of the whole liver, whereas liver biopsy only evaluates a small portion of the liver. However, liver MRI including PDFF in young children may require sedation with additional examination time and cost. Therefore, more convenient and cheaper diagnostic tests are required for screening and disease monitoring in patients with NAFLD, especially in children.

TE is widely used to evaluate liver elasticity and has been validated in large cohort studies for diagnosing and staging liver fibrosis [24]. CAP calculates the attenuation of ultrasonic signals acquired by TE, postulating that ultrasound propagation is affected by fat tissues on the path. CAP has been shown to have an excellent correlation with actual liver fat percentage in non-to-mildly obese patients with NAFLD [25] and can distinguish the absence or presence of steatosis in adult chronic liver disease [26, 27]. However, only one study with children has demonstrated the ability of CAP to detect steatosis, although differentiation among histopathologic grade of steatosis was not successful with small number of patients and fair overlap [14]. In that study, the suggested cutoff value of CAP for predicting steatosis was 225 dB/m with 87% sensitivity, 83% specificity, and an AUC of 0.93, which is comparable to the cutoff of 241 dB/m derived from our ROC analysis. In another study assessing CAP compared with ultrasound grading and the other imperfect gold standard in children, a cutoff point of 249 dB/m for predicting steatosis was identified with a sensitivity of 72% and a specificity of 98–100% [28], which is also comparable with our result.

In a prospective adult cohort study with PDFF as the gold standard [5], the cut off values for PDFF ≥5% and ≥ 10% were 288 dB/m (AUC 0.80, 95% CI 0.70–0.90) and 304 dB/m (AUC 0.87, 95% CI 0.80–0.94), respectively. In this study, the authors have identified that demographical characteristics, such as high BMI and high prevalence of type 2 diabetes, may affect the accurate assessment of CAP. The portion examined with the XL probe, which is reported to show higher value of CAP than using M probe [29], is also different with our study. A recent individual patient meta-analysis of CAP for assessing hepatic steatosis with various etiologies has shown that CAP values were influenced by several covariates, including BMI and the presence of NAFLD or non-alcoholic steatohepatitis (NASH), and suggested cutoffs of 248 dB/m for grade 1, 268 dB/m for grade 2, and 280 dB/m for grade 3 steatosis [13]. However, in another study from 2016, evaluating both PDFF and CAP in adult NAFLD [12], the cutoff values for hepatic steatosis grades 1, 2, and 3 were 5.2, 11.3, and 17.1% for PDFF and 236 dB/m, 270 dB/m, and 302 dB/m for CAP, respectively. The cutoff values for CAP and PDFF were both different in our study. Moreover, discrimination among steatosis grades 1, 2, and 3 with CAP was suboptimal in the present study.

One possible explanation for the discrepancy in cutoff values is that the histopathologic nature of pediatric NAFLD is different from that of adult disease. NAFLD patterns are characterized by a zone 1 distribution of steatosis, inflammation, and fibrosis in young children in contrast to the most intense change around the central vein (predominantly in zones 2 and 3) in adults [30]. This different pathologic distribution might affect the result of CAP on the basis of ultrasound technology. Other possible reasons could be the higher BMI and higher AST/ALT of patients in the present study, which could affect CAP results, as demonstrated in a study in adults [13]. Indeed, CAP values positively correlated with AWT (r = 0.517, *p* < 0.001), while PDFF did not. It could be from the difference of measurement way as PDFF measures a proportion of fat molecules and CAP measures physical properties of the liver. Whereas PDFF only evaluate liver parenchyma to separate fat and water signal, ultrasound signal on TE and CAP has no choice but to pass through the subcutaneous fat layer between TE probe and liver parenchyma. It is possible that the subcutaneous fat layer might affect the CAP measurement increasing ultrasound attenuation, especially in high BMI patients with increased AWT. A recent systematic review evaluated the factors affecting liver stiffness measurements using TE [31] and waist circumference was included as an affecting factor which is considered to be the same context as the CAP and BMI of this study. In addition, gender was included as covariate since different adipose distribution by gender might affect the result of CAP. In the present study, males had significantly higher AWT than females did, contrary to the previous study showing markedly higher subcutaneous thickness in females [32, 33]. Significantly higher rate of obesity and metabolic syndrome in Korean boys than in girls [3, 4] and small number of female patients (*n* = 24) in this study might affect the discrepancy. Additional studies with histopathologic correlations and analysis by gender will be needed to validate the effects of pathologic differences and AWT on CAP.

There are intrinsic limitations of both TE and MRI for the evaluation of NAFLD. Neither imaging modality can reliably discriminate NASH from simple steatosis [34]. A wide range of optimal cutoffs for the diagnosis of NASH has been reported and likely depends on the prevalence of advanced fibrosis in the study population [34]. In addition, current imaging methods cannot detect the lobular arrangement of steatosis, which is useful to distinguish the pediatric pattern of NAFLD [35].

This study also has several limitations. First, this was a retrospective study that included patients suspected with NAFLD who were referred by a pediatrician; thus, our results may have been affected by selection bias. Second, due to the lack of liver histopathologic data, we were unable to directly correlate and compare MRI and TE values with histologic grades of hepatic steatosis. Although PDFF based on MR imaging has demonstrated good correlation with histological steatosis grade, there were considerable overlap in PDFF results among steatosis grades [6, 7]. Therefore, histologic evaluation is still needed to determine the effects of simple steatosis and steatohepatitis on CAP values. Third, the number of patients, especially low proportion of the S0 group (*n* = 10), might be small to determine the optimal cutoff point, resulting in uncertainty of estimated cutoff value. However, in the retrospective study only including clinically suspected NAFLD patients, this is a reasonable result because there is no need to perform PDFF and CAP, without suspicion of NAFLD in pediatric patients. Moreover, the number of female patients is not enough for additional analysis by gender. Fourth, because there were no agreed criteria for severely obese patients in pediatric field, a BMI30 cutoff was applied. Nevertheless, the proportion of the BMI30 group was still low, so a further study to focus on severely obese patients is required.

## Conclusions

CAP can differentiate between the presence and absence of hepatic steatosis using a cutoff value of 241 dB/m with a sensitivity of 98.7% and a specificity of 80.0% in pediatric patients with NAFLD. However, CAP was not reliable in evaluating higher grade steatosis. Moreover, caution should be exercised in interpreting CAP data in obese children, especially in children with a BMI greater than 30 kg/m^2^, from the effect of increased AWT.

## Abbreviations

ALT: Alanine aminotransferase
AST: Aspartate aminotransferase
AUC: Area under the curve
AWT: Abdominal wall thickness
BMI: Body mass index
CAP: Controlled attenuation parameter
IDEAL-IQ: Iterative decomposition of water and fat with echo asymmetry and least-squares estimation quantification
NAFLD: Non-alcoholic fatty liver disease
NASH: Non-alcoholic steatohepatitis
PDFF: Proton density fat fraction
ROC: Receiver-operating curve
ROI: Region of interest
SSFSE: Single shot fast spin echo
TE: Transient elastography
